# A New Jam Factory

**Published:** 1901-07-27

**Authors:** 


### A NEW JAM FACTORY.

Mr. W. P. Hartley, who has carried on the jam-making industry at Aintree, Liverpool, for the last thirty years, has just opened a factory in Bermondsey. He was led to do this, he said on Tuesday, 25th ult., at the opening of the new premises, by the large demand for his jams and marmalades. The new factory is substantially built, the engines are in duplicate in case of accident; the boilers are of 1,200 horse-power, and every appliance had been made use of to reduce the smoke. Electricity will be used throughout for light, power, ventilating fans, etc., and labour-saving appliances have been designed to give the minimum of labour and to avoid the carrying of heavy weights.

Mr. Henry C. Cust, M.P. for Bermondsey, and the Mayor of Bermondsey, both of whom took part in the opening ceremony, owned to having felt very doubtful as to the wisdom of adding to the already crowded state of Bermondsey. They said they had, however, become convinced that a man with a record like Mr. Hartley's should be heartily welcomed. He cared for the health and social well-being of the workpeople, and at Aintree he had introduced the profit-sharing principle — £28,600 had been distributed during the last thirteen years in this way alone. In the London works every consideration had been shown for the workpeople's health, and the Mayor added that possibly Mr. Hartley might devise some means for housing his employés, as he had done at Aintree. The housing of the people was a question he said which, as Mayor, he had to consider.

About 700 persons will be employed at the new works, the majority being women and girls. Although jam-making from fresh fruit is a season-trade, they will be employed during the rest of the year in the making of pickles, to the manufacture of which one building will be devoted.

